# Validation of 4D Components for Measuring Quality of the Public Health Data Collection Process: Elicitation Study

**DOI:** 10.2196/17240

**Published:** 2021-05-10

**Authors:** Hong Chen, Ping Yu, David Hailey, Tingru Cui

**Affiliations:** 1 School of Computing and Information Technology Faculty of Engineering and Information Sciences University of Wollongong Wollongong Australia; 2 Jiangxi Provincial Centre for Disease Control and Prevention Nanchang China; 3 Illawarra Health and Medical Research Institute University of Wollongong Wollongong Australia; 4 School of Computing and Information Systems University of Melbourne Melbourne Australia

**Keywords:** data quality, data collection, HIV/AIDS, public health informatics, health information systems, component validation, expert elicitation, public health, health informatics

## Abstract

**Background:**

Identification of the essential components of the quality of the data collection process is the starting point for designing effective data quality management strategies for public health information systems. An inductive analysis of the global literature on the quality of the public health data collection process has led to the formation of a preliminary 4D component framework, that is, data collection management, data collection personnel, data collection system, and data collection environment. It is necessary to empirically validate the framework for its use in future research and practice.

**Objective:**

This study aims to obtain empirical evidence to confirm the components of the framework and, if needed, to further develop this framework.

**Methods:**

Expert elicitation was used to evaluate the preliminary framework in the context of the Chinese National HIV/AIDS Comprehensive Response Information Management System. The research processes included the development of an interview guide and data collection form, data collection, and analysis. A total of 3 public health administrators, 15 public health workers, and 10 health care practitioners participated in the elicitation session. A framework qualitative data analysis approach and a quantitative comparative analysis were followed to elicit themes from the interview transcripts and to map them to the elements of the preliminary 4D framework.

**Results:**

A total of 302 codes were extracted from interview transcripts. After iterative and recursive comparison, classification, and mapping, 46 new indicators emerged; 24.8% (37/149) of the original indicators were deleted because of a lack of evidence support and another 28.2% (42/149) were merged. The validated 4D component framework consists of 116 indicators (82 facilitators and 34 barriers). The first component, data collection management, includes data collection protocols and quality assurance. It was measured by 41 indicators, decreased from the original 49% (73/149) to 35.3% (41/116). The second component, data collection environment, was measured by 37 indicators, increased from the original 13.4% (20/149) to 31.9% (37/116). It comprised leadership, training, funding, organizational policy, high-level management support, and collaboration among parallel organizations. The third component, data collection personnel, includes the perception of data collection, skills and competence, communication, and staffing patterns. There was no change in the proportion for data collection personnel (19.5% vs 19.0%), although the number of its indicators was reduced from 29 to 22. The fourth component, the data collection system, was measured using 16 indicators, with a slight decrease in percentage points from 18.1% (27/149) to 13.8% (16/116). It comprised functions, system integration, technical support, and data collection devices.

**Conclusions:**

This expert elicitation study validated and improved the 4D framework. The framework can be useful in developing a questionnaire survey instrument for measuring the quality of the public health data collection process after validation of psychometric properties and item reduction.

## Introduction

### Background

Public health, a data-intensive discipline, relies on high-quality data to monitor the health and well-being of the population, make appropriate policy decisions for intervention, and evaluate intervention outcomes [1-3]. After two decades of development in the design and implementation of information and communication technologies (ICTs) for national public health data management, public health information systems (PHISs) have developed into essential data repositories [1,4,5]. PHISs have been well integrated into many nations’ health information management systems, such as those of the United States, Australia, and China [6-9]. The data stored in PHISs, for example, on women’s and children’s health, aging population, and people living with HIV/AIDS, have enabled public health agencies to formulate evidence-based policies and plan and evaluate program performance to ensure accountability for citizens and countries [1,6,7,10].

As data-driven public health management assumes data are accurate, timely, and reliable, data quality assessment needs to be continuously and rigorously conducted to ensure high-quality data in PHISs [4]. Data quality is a 3D concept that includes the quality of data, data collection process, and data use. Improving the quality of the data collection process is critical for PHIS data quality management [11]. Identification of the essential components of the quality of the PHIS data collection process is the starting point for the design of effective public health data quality management strategies [4,7].

Through appraisal and synthesis of literature that reports the factors affecting the rigor of the PHIS data collection process, we have proposed a preliminary conceptual framework that focuses on four dimensions of the quality of the process [12]. These are data collection management, data collector, information system, and the data collection environment. We name them 4D components, which consist of 12 subcomponents and 149 indicators (Multimedia Appendix 1 [7,12-15]). Data collection management is an administrative process by which data are acquired, validated, stored, protected, and processed [7,13]. Its indicators include appropriate data collection methods, data entry forms, and ongoing quality assurance. At the individual level, data collection personnel (replacing *data collector*) need to have a right attitude, adequate skills, and competence for the job. They must maintain adequate communication with each other. For them to execute their tasks adequately, their organization needs to provide adequate staffing with the right skill mix [12]. A data collection system (replacing *information system*) requires different systems and elements to integrate and assist data capture, data entry, and data logging. Thus, continuous and systemic functionality and technical support are required [14]. A good data collection environment includes training, strong leadership, and funding support for data collection [15]. Given that this preliminary 4D component framework was derived from an inductive analysis of the literature, validation of the framework within a certain PHIS was needed.

Expert elicitation is a research method used to identify and address uncertain subjects, especially when relevant local evidence or information is incomplete [16]. This method has been widely used in public health for policy decisions to generate evidence [17,18] to achieve various research goals, such as environmental health impact assessment [16], health technology assessment [19], and economic evaluation of health gains of antenatal care [20]. Knowledge synthesized from expert opinions can form the foundation for further research.

The main procedures for a formal expert elicitation include characterization of uncertainties, selection of experts, elicitation of expert judgments, and possible aggregation and reporting in a temporary summary [16]. The criteria for expert selection include the following: the person should be representative of the main population of interest and he or she should have sufficient intellectual ability to provide the theoretical definitions, rank the importance of the data items, and explain a potential causal relationship between them [16]. Expert judgments should adhere to the principles of the scientific process. These are accountability, neutrality, fairness, and the ability for empirical control [21]. A facilitator, often a trained interviewer, has *the enormous potential* *to reduce bias in expert elicitation* by clarifying the questions to the expert [16,19]. A systematic elicitation session could increase the validity, transparency, and trustworthiness of research [16].

### Objectives

Using an expert elicitation approach, this study aims to obtain empirical evidence to confirm the components of the 4D framework and, if needed, to further develop the framework.

## Methods

### Study Setting

The study was conducted within a country-level PHIS, the Chinese HIV/AIDS Comprehensive Response Information Management System (CRIMS). Acknowledged as one of the milestones for China’s HIV/AIDS response programs over the past three decades [22], the CRIMS is a web-based national AIDS information management system that was established in 2008 [5]. The system has been used for routine HIV/AIDS prevention and control data collection from hospitals and all units of the Chinese Center for Disease Control and Prevention (China CDC) in 2893 counties. The data stored in the CRIMS include demographic information, case reporting, antiretroviral treatment, methadone maintenance therapy, behavioral interventions, laboratory testing, counseling, and surveillance. These real-time data can be used for decision making, monitoring, and evaluating HIV/AIDS prevention and control programs in health bureaus and CDCs at national, provincial, city, and county levels [10]. Therefore, high-quality data in the system are imperative for China’s HIV/AIDS program monitoring and evaluation. However, prior studies found that public health professionals lacked trust in the quality of data in the CRIMS and expressed concerns over the quality of the data collection process [17,23,24]. This primary concern of public health professionals in China has also motivated this study.

Data management within the CRIMS includes data collection, data entry, data analysis, data assurance, and data use [25]. The personnel involved in the CRIMS data management include health administrators in health bureaus, epidemiologists and laboratory technicians in CDCs, and clinicians and data registrars in hospitals. They have accumulated rich experiences from long-term empirical work in HIV/AIDS data management and were thus appropriate experts who could provide inputs for this study.

### Ethical Approval

This study was approved by the Human Research Ethics Committee at the University of Wollongong and the Institutional Review Board of the National Center for AIDS/STD Control and Prevention at the China CDC. All participants provided informed written consent to participate in the study and to publish individual data.

### Design of Interview Guide and Data Collection Form

To ensure the validity of the study, we followed three broad categories of validity for qualitative research in information systems proposed by Venkatesh [26]. These are (1) design validity (eg, descriptive validity, credibility, and transferability), (2) analytical validity (eg, theoretical validity, dependability, consistency, and plausibility), and (3) inferential validity (eg, interpretive validity and confirmability).

During the design phase, an interview guide was developed in consultation with 7 information system researchers at the University of Wollongong: a professor, an associate professor, a lecturer, a research assistant, and 3 PhD candidates. Two open-ended questions were suggested: “What are the components of quality of the CRIMS data collection process?” and “What are the attributes of these components?”

An item represents a component or subcomponent of the 4D component framework in reference to the literature [12]. An item weight table was developed to elicit an expert’s opinion about whether an item is a component or subcomponent of the quality of the CRIMS data collection process. To avoid bias in directing the expert to the preliminary 4D component framework, we reconstructed the testing items according to group discussions with consultant researchers. Four items that are not part of the framework but frequently identified in practice were added, including parallel organization, high-level management, social factors, and organizational policy. Four items that are elements of a certain original subcomponent or component were used to represent their parental components. These were data collection forms and data quality assessment strategies of the component data collection management, data collector’s data quality audit skills, and demographics of the component data collection personnel. Four original items—funding, data collection personnel’s communication, staffing pattern, and integration of different systems—were purposely excluded to test the completeness of the framework item spectrum. Each item was answered as *yes* or *no*. If the answer was yes, the expert was asked to rank the importance of the item for the quality of the CRIMS data collection process. The rankings ranged from 1 (the highest contribution) to 16 (the smallest contribution; Multimedia Appendix 2).

The interview guide and item weight table were translated into Chinese. Three bilingual authors validated the Chinese translation. The interview guide was pilot tested for content validity and face validity with 8 Chinese public health practitioners who worked within the CRIMS. All 8 practitioners agreed with the fit of the interview questions and the item weight table for the study.

### Sampling and Recruitment of Study Participants

To ensure generalizability of the study, personnel from all administrative levels in all types of organizations with at least one data management role for the CRIMS were considered as potential experts. They were eligible for inclusion as experts to ensure a comprehensive capture of diverse expert opinions. Those who did not have a role in CRIMS data management were excluded.

Following the aforementioned selection criteria, we used a stratified sampling method to identify the participating organizations [27]. Representativeness was ensured by a lack of significant statistical differences in data quality among provinces [23,24]. A total of 19 organizations were selected, including 3 departments of health bureaus (1 each at the central, provincial, and county levels), 10 departments of the CDCs (1 each at the national, provincial, and city levels and 7 at the county level), and 6 hospitals (4 tertiary, 1 secondary, and 1 primary).

HC was an epidemiologist who specialized in HIV/AIDS prevention and control in a provincial CDC in China. She used a convenient sampling method to recruit participants working in health bureaus and CDCs. A personalized invitation message containing a cover letter and a consent form was sent through the Chinese social media QQ to 20 potential participants. All participants agreed to participate by returning a completed consent form. Web-based interviews were arranged with 18 of them through QQ media. The other 2 withdrew quoting time constraints. Of the 18 participants, 3 were from health bureaus at 3 different levels. The remaining 15 came from 4 tiers of the CDCs: 1 at the national level, 4 at the provincial level, 3 at the city level, and 7 at the county level.

HC recruited potential participants from 6 hospitals via direct contact with hospital management. She explained the project’s purpose and research process to the relevant managers in the hospitals and sought their support in recommending eligible data management personnel to participate in the field study. Being introduced by the facility management, HC contacted the potential participant and organized an interview with the person at a designated venue and time. An interview would start only after providing written consent. Overall, 10 potential participants were recommended and completed interviews. Of the 10 participants, 6 came from 4 tertiary hospitals, 3 from a secondary hospital, and the other from a primary health care center.

On average, the 28 participants had worked in public health or health services for 12 (SD 7) years and in the HIV/AIDS domain for 7 (SD 4) years. Of the 28 participants, 16 (57%) were female; 23 (82%) participants were aged between 30 and 50 years, and the other 5 (18%) were aged under 30 years. Most participants (25/28, 89%) had multiple job roles in HIV/AIDS data management.

### Interview Procedure

Both telephone and face-to-face interviews were conducted. An internet voice call was made for telephone interviews with the practitioners during their work break or after hours. Face-to-face interviews were conducted at hospitals. The average duration of the interviews was 44 minutes (SD 23 min).

Each interview started with asking the practitioner to provide answers to the 2 open-ended questions. Answers from 3 of the first 5 practitioners were related to data quality instead of the focused topic of this study, the quality of the data collection process. To clarify the research topic, the researcher started subsequent interviews with the question, “What do you think the differences are between data quality and quality of the CRIMS data collection process?” A further probe clarified any emerging issues raised by the practitioners. Once information saturation was reached, that is, no further issues emerged, the interview was concluded.

After the practitioners answered all the open-ended questions, they were invited to assess the 16 items listed in the weight table. The other 7 items were raised by the practitioners. Their average rankings were not calculated because of the small sample size.

### Data Processing and Analysis

All audio recordings were transcribed verbatim. The transcripts were sent to the interviewees for confirmation, translated into English, and back translated. Qualitative data analysis was conducted in accordance with the framework analysis approach suggested by Pope et al [28]. The theoretical (thematic) framework was the 4D component of the quality of the PHIS data collection process. The unit of analysis was each transcript. The data analysis was conducted in 3 steps.

#### Step 1: Generating the Initial Codes

Each transcript was thoroughly read to identify and understand the meaning of the relevant text. A concise phrase was created to summarize a sentence. For example, “Reward and punishment system, which is important for a working system...This should be in organizational management policy” (C102) was coded as “clear reward and punishment in organizational policy.” “If they (managers) understand the importance to the job (data collection), you will work easily; if they don’t, it is hard” (H306) were coded as “managers should understand the importance of data collection.”

After the first round of transcript encoding, 302 codes were extracted and stored in an Excel database.

#### Step 2: Mapping the Codes Using the Preliminary 4D Component Framework

All the 302 codes were compared and mapped with the original indicators and subcomponents in the preliminary framework. Three data processing strategies were used in 3 different scenarios.

##### Scenario 1

When a code had a similar meaning to an original subcomponent or indicator of the preliminary 4D framework, the original subcomponent or indicator remained or was further refined by merging, condensing, and grouping to represent the code.

##### Scenario 2

When the meaning of a code was not matched by any original subcomponent or indicator in the preliminary 4D framework, a judgment was made to add the code as a new subcomponent or indicator to the framework.

##### Scenario 3

When no empirical data could match the meaning of a certain subcomponent or indicator in the preliminary 4D framework, the subcomponent or indicator was deleted from the framework.

Iterative and recursive coding, mapping, and classification processes were applied continuously between steps 1 and 2. The 302 codes converged to the 4D component framework; 88 were grouped into the component data collection management, 86 into the data collection environment, 77 into the data collection personnel, and the remaining 51 into the data collection system. A total of 46 new indicators emerged from the extracted codes. Of the 149 original indicators, 37 (24.8%) were deleted because of a lack of evidence support and 42 (28.1%) were further merged with codes with similar meaning but different wording. Finally, 116 indicators, 16 subcomponents, and 4 components were synthesized.

#### Step 3: Interpreting the Framework

**Figure 1:**
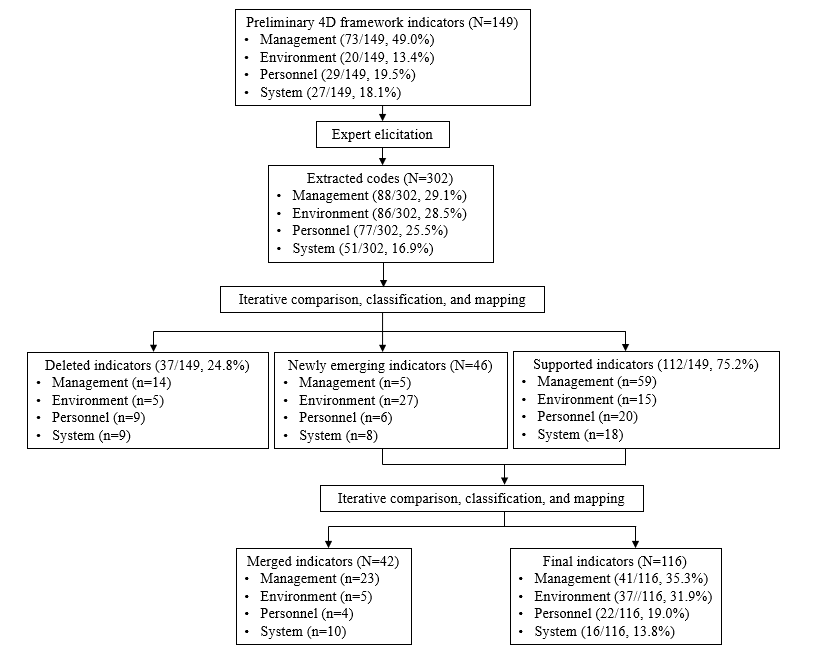
The validation process for the 4D framework.

The nature of and associations among the components, subcomponents, and indicators were further assessed by the author group. Each indicator was identified as either a facilitator or a barrier for data collection. Data and themes that had been extracted from expert elicitation were constantly compared between hospitals and CDCs with varying data collection processes and contexts and between different data collection roles played by different experts. The data analysis outputs were triangulated and discussed within the team until a consensus was reached (Figure 1).

## Results

### Overview

**Figure 2:**
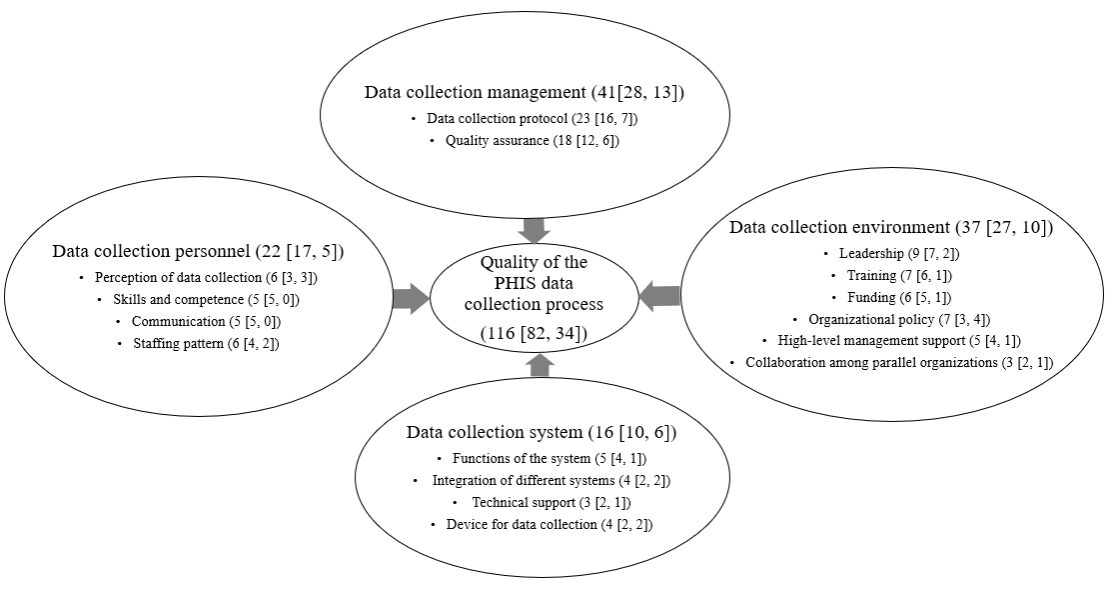
Composition of the 4D framework. Parenthesis: (number of indicators [number of facilitators, number of barriers]). PHIS: public health information system.

The 4 dimensions of the 4D framework were verified as data collection management, data collection environment, data collection personnel, and data collection system. Three new subcomponents were added: organizational policy, high-level management support, and collaboration among parallel organizations. A total of 16 subcomponents were validated and grouped into the appropriate 4D components. They were measured by 116 indicators, including 82 facilitators and 34 barriers (Figure 2).

The next section presents the results using the 4D components to tabulate and elaborate the evidence that supports the subcomponents and indicators of the validated 4D framework situated in the CRIMS.

### Data Collection Management

Data collection management includes 2 essential subcomponents: *data collection protocol* and *quality assurance*. Of the 302 interview codes, 88 (29.1%) supported 59 original indicators of data collection management. The remaining 14 were deleted because of a lack of evidence support. Furthermore, 5 new ones emerged from the interview codes. After merging 23 supported original indicators to amalgamate similar meaning with different wording, 41 indicators, including 28 facilitators and 13 barriers, were finalized for measuring the data collection management (Multimedia Appendix 3).

#### Data Collection Protocol

A total of 56 interview codes were related to the subcomponent *data collection protocol*. They validated 23 indicators, including 16 facilitators and 7 barriers, and fell under the subdimension of data collection form and data collection methods.

Six practitioners (C302, C303, C201, C101, C106, and A101) suggested that the data collection protocol should be aim-focused, operable, scientific, rational, and feasible for frontline data collectors. It should contain comprehensive, step-by-step guidance for the entire process of data collection (A101 and C201). The involvement of frontline data collectors in the development of a data collection protocol was an optimal practice (C302).

A total of 16 practitioners (C101, C102, C103, C104, C105, C106, C107, C201, C203, C303, C304, A101, H302, H305, H101, and H202) stressed that a data collection form needs to be clear, readable, comprehensive, and unambiguous. One of the practitioners mentioned:

It [design of the form] needs to be rational to make data collection convenient and simple, and provides comprehensive data, should reduce data collectors burden and reduce unnecessary effort.

C102

To ensure that the questions about data collection are articulated in a scientific, rational, and operable manner, 3 CDC practitioners (C201, C202, and C107) recommended the following: (1) to solicit a question, one can ask questions from different angles; (2) the number of questions should be suitable and controlled within the allotted data collection time; (3) the wording of the questions, including options for the multiple-choice questions, must be accurate, direct, understandable, and answerable; and (4) questions should be bound within ethical considerations and should not cause harm to respondents, particularly in places where it is challenging to find confidential and private space for question elicitation.

The data collection methods should be well developed, uniform, applicable, and implementable for data collectors (C302 and C301). A method is considered optimal for data collection if the task is integrated into routine data flow in a health care facility.

#### Quality Assurance

Overall, 32 interview codes were related to the subcomponent *quality assurance* and validated a total of 18 indicators, including 12 facilitators and 6 barriers. Three topics were elicited: the criteria of quality assurance, the constituency of quality assurance, and the implementation of conduct of quality assurance.

The criteria of quality assurance were consistent with the requirements of data quality, that is, accuracy, completeness, and timeliness. Therefore, quality assurance is “able to thoroughly, quickly and accurately assess data accuracy, completeness and timeliness” (C203).

Clinicians believed that data quality audits were useful in motivating data collectors because their managers may provide extra funding to incentivize this activity. H201 explained the advantage of a data audit:

On one side, it is useful to provide further verification guidance to our routine work, and correct deficiencies inevitable in operational procedures because we are new to this job. On the other side, if they could brief the findings to our manager, it would be even better...For example, if my workplace was equipped with the needed amenities, then it will be easy and convenient. It does not necessarily need further monetary injection.

H201

Two health administrators (A401 and A301) who held a position at the national and provincial administration used the CRIMS data regularly for decision making. They relied on the quality of the data quality assessment conducted by all levels of CDCs. “The professionals will ensure the quality of data collection process” (A401), whereas “it is impossible to verify the situation (of data) in the front line” (A301).

A401 expressed his concern about the deliberate falsification of data, especially the *soft* data. Soft data means that its data quality is difficult to assess even with field verification, such as data from high-risk population intervention, follow-up, and health education. *Hard* data are more likely to be *true*, for instance, the methadone treatment data documented on the site, and thus, *hard* data are less prone to errors:

It does have difference in level of data accuracy. Some data are relatively accurate, such as the data about methadone treatment because they were recorded when the patient took the medicine; that possibly would not be falsified, right? However, intervention data, the “relatively soft data”, are hard to verify in the office. If you do not make an on-site verification, it is hard to control the recording of them.

A401

### Data Collection Environment

The data collection environment includes 3 original subcomponents (leadership, training, and funding) and 3 newly added ones (organizational policy, high-level management support, and collaboration among parallel organizations). Of the 302 extracted codes, 86 (28.5%) were about the data collection environment, with 32 relating to the 3 new subcomponents. A total of 27 new indicators emerged from these interview codes. Of the 20 original indicators, 5 were deleted because of a lack of evidence and another 5 were merged further for a similar reason. A total of 37 indicators, including 27 facilitators and 10 barriers, were finalized to measure the data collection environment (Multimedia Appendix 3).

#### Leadership

Of the 28 practitioners, 26 (93%) agreed that leadership is a subcomponent of the quality of the CRIMS data collection process, ranking first among all subcomponents. Twenty-four codes were related to *leadership*. A total of 9 indicators, including 7 facilitators and 2 barriers, were validated for measuring leadership.

Concerning qualification and role, leaders should be role models with professionalism (C103, C105, C106, C203, and H304). They are **“**able to ensure the procedures to be executed up to standards, ensure the implementation of requirements and protocols of data collection, analysis, and use, and thus ensure data quality” (C203). To initiate a new task data collection, the leaders should have a clear roadmap for assigning and executing the task (A101). Leaders should have strong organizational capabilities to push it forward (H301, H303, and C106). Therefore, leaders do not necessarily have to do everything by themselves but should be familiar with the task requirements (C103, C104, and C105). They should have the power to issue policies, clarify and assign duties and tasks, and provide financial support (C302 and C104).

The extent to which a leader attaches importance to data collection determines the quality of this task. “People follow the example of their superiors” (H304 and H305). Clinicians (H202, H203, H301, and H306) mentioned that a significant indicator of adequate notice by a leader in charge is the frequency of his or her attending the meetings or the supervision and inspection events organized by the CDC.

From the practitioners’ perspective, a good leader could “lead us well, ensuring those willing to do to have the opportunity to do, and turn those reluctant to do into willing to do; this is good leadership” (H304). The management recognition of the contribution of data collection personnel to data quality is an important motivation factor for data collectors (C102 and H305). It could be in the format of “oral praise to recognize and formal acknowledgement beyond financial incentives” (C102). As commented by H305 and H306, “our leaders all think highly of this job (data collection). Otherwise, the staff would not care.” “Data collection personnel need to be respected, trusted, acknowledged, and complemented by leaders” (H304, C104, and C302).

#### Training

Of the 28 practitioners, 27 (93%) agreed that training is a subcomponent of the quality of the CRIMS data collection process, ranking second among all components. A total of 19 interview codes about training generated and validated 7 indicators, including 6 facilitators and 1 barrier focusing on the objective of training, and the methods to deliver it and evaluate it.

The goal of training is to equip data collectors with qualified work competence and professionalism (C102, C103, C104, C105, C106, and C302):

The training objective is to equip the data collectors with work competence, with good work professionalism, such as work abilities and skills.

C302

I think training is more related to communication of [data collection] skills. Firstly, we must be familiar with the survey, then we will explore how we get good data. Learning skills is an objective to be reached via training.

C104

Therefore, training needs to focus on the standardization and uniformity of the data collection process. These include objective, methods, and time frames for data collection (C203, C103, C104, C106, and H304). The trainees should understand the definition of data to be collected, have good knowledge about all procedures for data collection, and adhere to the standardization.

Interactive training between trainers and trainees has been suggested (C103). During training, trainers should address work issues and help trainees learn what to do and how to do it (C103 and C105). Trainers should not just talk and go and be disinterested in whether the trainees understand or not. Trainers who were welcomed by trainees were those who quickly responded to trainees’ questions (C105) and those providing empirical field practice examples in the training session. C103 suggested “if the trainers give more empirical examples for the training, the trainees may obtain a better understanding.”

Data collection personnel, especially the newly recruited staff, need training after recruitment and refresh their knowledge every year about what and how to do. On-the-job training, hospital webpage training, and exams have been used in health facilities (H101 and H306). Building up a training network that installs materials and sources under the circumstance of high staff turnover is recommended (C106).

Given that the training results might vary among trainers, a training assessment was recommended, including selecting trainers, training methods, and training contents. C103 claimed that the higher the level of a training organization, such as international organizations and high-level CDCs, the better the training quality.

#### Funding

Although the subcomponent funding was not included in the item weight table, 10 relevant codes emerged from the interview transcripts and generated 3 new indicators. Three original indicators remained. They gave rise to 5 facilitators and 1 barrier to measuring the subcomponent funding.

From the CDC professionals’ perspective, funding should support purchasing data collection devices such as computers, printers, and even vehicles (C301, C103, and C104). Funding should provide compensation, such as gifts for health clients to seek their cooperation (C103). Otherwise, “without funding support, without policy, and without competent personnel, data quality may be problematic, or even a fake product made up in office” (C103). Continuous funding support for previously funded projects is needed to avoid the situation of “when the Demonstration Project finished, funding decreased significantly” (C104).

From the hospital data registrars’ perspective, HIV/AIDS work does not bring in profit, an activity that does not support the profit goal of the hospital (H301, H202, and H203):

HIV/AIDS prevention activities do not bring in profit, the doctors in the hospital should be committed and have spirit of dedication. However, in market economy, hospital needs profit, and is focused on pursuit of economic cost effectiveness.

H301

Without funding support, clinicians think they are volunteers for HIV/AIDS data collection. Therefore, sometimes, they are unwilling to do this job.

H203

Therefore, given that “funding support can provoke work” (H201) and “the cost of management and treatment can be reimbursed” (H203), “funding support for data collectors must be fully implemented” (H202 and H203). The health administrator (A101) had already recognized this need and promised to further push this agenda:

In another aspect, it might be related to boosting work morale to encourage them [data collection personnel] by increasing funding support. For example, they may get some subsidies for the work they are doing or have done. Currently we do have some funding. The performance-based salary system is inflexible. It may be problematic to link their income with their performance. This shortage, maybe, is what we need to tackle, for example, from the perspective of national management. We should be able to do, but not much has been done yet. They should get a better income. This aspect is what we should do.

A101

#### Organizational Policy

Organizational policy is a new subcomponent. Of the 28 practitioners, 23 (82%) agreed to place it in the component of the data collection environment. A total of 13 codes were related to the organizational policy and generated 7 indicators, 3 facilitators and 4 barriers. These indicators were primarily concerned with what organizational policy is desirable for HIV/AIDS data collection.

The organizational policy was critical to ensure the execution of the data collection activities (C104 and H101). “If they attach importance to the job, you will work easily; if they don’t, your work is a challenge” (H306). It was regarded as more important than the actual process of data collection because the latter was under the control of the data collector (H303). The organizational policy should “support recognition and reflection of the real situation and encourage analysis of existing issues, a proactive adaptation of scientific findings generated from analysis of high-quality data” (C203).

Desired organizational policies of the CRIMS data collection process included (1) ensuring sufficient funding, staffing and material support, for example, “as long as the workload is increased, more staff is assigned” (H101); (2) embodying good management and coordination; (3) having a built-in reward and bonus scheme (C301 and C202) to “motivate people to work well” (H303).

Indicators of a poor organizational policy relating to data collection included the following aspects: (1) data collection was set up as a part-time job, (2) narrow workspace insufficient for data collection (H302), (3) increased workloads without adequate payment (H201), and (4) the culture of “eating big-pot-rice” (C106).

#### High-Level Management Support

High-level management support was another newly added subcomponent that 79% (22/28) of the practitioners agreed to. A total of 19 interview codes generated 5 indicators, including 4 facilitators and 1 barrier, to measure this subcomponent of high-level management support.

High-level management support provides assurance (C201); assistance for training; responsibility for policymaking (H305); and being scientific, specific, and rigorous for decision making (C104 and C106). It enforces an appropriate reward and punishment mechanism (H303). High-level management support also means funding support and making essential data collection tools such as vehicles available (C103).

Conversely, high-level management support should “not impose excessive administrative pressure on data collectors because it may compromise data integrity and accuracy in data collection. The management should not affect and intervene in the data collection process and the data. Otherwise, it may cause a negative consequence of manipulating results” (C203). In practice, the policy had a significant impact on the data quality (C104 and C302). The health administrator (A301 from the provincial health bureau) had a different viewpoint: “currently, as for the HIV/AIDS epidemic data collection, indeed there is no intervening in our work, basically it (data) is reliable, no concealment.”

High-level management support was recognized as “a strong power that can veto by just a couple of words” (A101 from the county health bureau). The more the emphasis on data quality placed by upper management, the more time would be invested by data collectors toward data quality and vice versa (C102). “No site auditing, no proper work” (H303). However, the more the layers between the high-level management and the frontline data collection organization, the more difficult it is for the organization to execute the data collection process (C104).

The CDCs were considered by H201, a clinician in a secondary hospital, as “supportive” high-level management; they were expected to provide hospitals with support and advocacy. The CDCs were also expected to be of help and to understand *“*why, what and how” about data collection. H301, a data registrar in a tertiary hospital, suggested that the local CDC should “clarify the work-flow in hospital and do not just require us doing this and doing that without distinction.”

#### Collaboration Among Parallel Organizations

Collaboration among parallel organizations was a third newly added subcomponent, with 82% (23/28) practitioners agreeing. A total of 14 interview codes were related to this subcomponent, which may contribute to HIV/AIDS data collection, for example, through hospitals and CDCs. Furthermore, 3 indicators, including 2 facilitators and 1 barrier, were added to the 4D framework to measure collaboration among parallel organizations.

It was found that sometimes the quality of the data collected by the collaborating organizations may have inferior quality to those collected by the CDCs, if they are without staff in charge. Therefore, if data to be collected were provided by a collaborating organization, C403 suggested a coordinating HIV/AIDS committee would contribute to high-quality data collection. He stressed,

“If the parallel organizations with dependency in data do not have a right attitude toward data collection, or conduct data collection in a reckless manner, then the data to be collected would be worse (in quality) and useless.

C403

A public health professional (C203) working at a city-level CDC stated that the parallel organization “should not use vicious competition and negative approaches to intervene with public health data collection and organizations. They should cooperate, coordinate and facilitate.”

### Data Collection Personnel

The component *data collection personnel* included 4 essential subcomponents: *perception of data collection, skills and competence, communication*, and *staffing patterns*. Of the 302 interview codes, 77 (25.5%) supported 20 of the 29 original indicators of the data collection personnel in the preliminary framework. Six new indicators emerged, and 4 were merged further. There were 22 indicators, including 17 facilitators and 5 barriers for measuring data collection personnel.

#### Perception of Data Collection

All 28 practitioners agreed that data collectors’ perception of data collection is an important subcomponent determining the quality of the CRIMS data collection process. Of the 6 original indicators about the perception of data collection, 4 were supported by the interview transcripts, 1 was deleted because of a lack of evidence, and the other was merged with 2 newly added indicators. Six indicators, including 3 facilitators and 3 barriers, were finalized to measure the perception of data collection.

From some practitioners’ perspectives (C102, C103, H306, H202, and H203), the CRIMS data collection process would not be as complicated if the data collection personnel were aware of its importance, which would also lead to better data quality. As H203 said*:*

It is a matter of how serious they (doctors) are definitely. Because this (data collection) is a very simple and easy job. If you pay attention to it, you can do it well.

H203

H202 and H203, 2 public health data registrars working in a secondary hospital, agreed that the priority given by clinicians and managers in the hospital could significantly improve the quality of the data collection process and thus data quality:

It is an issue of whether the doctors and management value it (data collection). If the management values data collection, doctors will also value the activity.

H203

It was suggested that the *perception of data collection* should not only be measured by receptibility to data collection but also by 2 new indicators, including commitment of the data collection personnel to data collection and their attitude to integrity (C103, C201, C203, C302, and H203). The manifestation of *good* attitude may be “data were consistent between the paper-based and the electronic records of the CRIMS” (C103). The fabrication of data or negligence is often caused by poor attitudes rather than incompetence or lack of training for data collection. Burnout demotivates data collection personnel to treat the job as their job responsibility. C106, a public health professional with 8 years of work experience at a county CDC for HIV/AIDS prevention and control, suggested that burnout may appear after working on the same job for a long period. “Now nobody values much about this job, so not many are willing to do it, including me” (C106).

#### Skills and Competence

All 28 practitioners agreed that data collection skills and work competence were important for data collection personnel. Five indicators, all facilitators, were recommended for measuring subcomponent skills and competence.

This subcomponent was a *must-have* capability for frontline data collectors (C202), which is more important than the data collector’s education level (C201, C102, and C103):

If they [with high education degree] do not have adequate work experience, if they do not have work skills, they cannot find the solution to the problem.

C201

Besides the skills for data quality check, the subcomponent skills and competence includes an accurate understanding of the objective of data collection, contextual knowledge, and the definition of data items (C102, C103, C106, and H102). Data collection personnel should be able to make a rational judgment about the reliability of a data source and ensure data accuracy and completeness (C302, C202, C203, C104, C105, C106, A101, and H302). Communication, organization, coordination, and writing skills were also desired skillsets recommended by practitioners for a competence-based framework (A101, C302, C201, C102, H302, and H305).

The data collection personnel should be professional and receive training in data collection. Interns were not considered qualified for data collection and reporting. H302, a clinician from a tertiary hospital, suggested that work competence means being mature and experienced, which is not what an apprentice is up to. H301 and H306 reported that the interns in tertiary hospitals were asked to fill in the data collection forms for busy clinicians.

#### Communication

Although communication was not listed in the item weight table, a lack of good communication among data collectors, as described in the preliminary framework with 5 facilitative indicators, was verified by the practitioners, particularly those who need to directly interact with health clients in routine work (H302, H305, H201, and C106).

H201, an HIV/AIDS clinical specialist, felt embarrassed in detecting transmission routes through conversations with AIDS patients. She thought that transmission routes were a private issue, especially for young men. If the data to be collected do not affect treatment, then data quality can be compromised in the interest of preserving the privacy and dignity of patients:

All in all, it (knowing whichever transmission route) does not affect treatment. Through conversation with them, I feel that these patients are worried about we, doctors, are discriminating against them. This is the major concern. So, collecting this type of data (transmission route) is neglected in my job.

H201

C106, a county CDC professional, felt that it was difficult to communicate with AIDS patients during follow-up:

Sometimes, I do not even know how to communicate with them. Like meeting someone new, I am not sure what kind of psychological characteristics the person has. Basically, I feel them difficult to deal with. I do not even know how to talk to them. Sometimes it is fine; this feeling has always been there.

C106

She also felt that she was not getting adequate support from her colleagues in a routine job:

Having been in this job so long, it is embarrassing to ask others certain problems you encounter. You can only formulate solutions by yourself. You find it difficult to ask others. Better do it yourself.

C106

#### Staffing Pattern

Although the staffing pattern was not in the item weight table, it was mentioned by 11 practitioners. A total of 18 interview codes supported 6 of the 7 original indicators, including 4 facilitators and 2 barriers.

Practitioners frequently mentioned a lack of an adequate number of competent public health professionals:

There are only two staff members assigned to work at the front line of HIV/AIDS control by the Department of HIV/AIDS. These two staff members have to collect all data, and they are under enormous pressure; this indicates the staffing level is inadequate. [C103]

I feel the most challenging is staffing level. Lots of work needs people to do. It does not mean there is no staff to do the work but almost everyone has several parallel lines of work happening at a given time. Like us, old employees, all part time regarding data collection.

C107

In C107’s workplace, employment of contractors was a major approach to fill the vacancy, but it was not favored by local public health professionals because of high turnover. The professionals even refused to train the contractors because they were worried that their efforts would be wasted if the contractors quit the job soon after the training was completed.

Experienced staff and female staff were considered (by C302, C201, C101, C106, and H302) to be the optimal personnel for collecting quality data, rather than young practitioners, because of their experience in interacting with and establishing rapport with AIDS patients. Four practitioners (C302, C105, C106, and H305) suggested that education level, training, experience, personality, and value could affect work competency and, thus, the quality of data collection.

The health administrator from the national Ministry of Health (A401) suggested a need to increase the recruitment of frontline data collectors to cope with the increased workload in HIV/AIDS prevention and control.

### Data Collection System

The component data collection system includes 4 subcomponents: functions of the system, integration of different information systems, technical support, and devices for data collection. A total of 51 codes for this component were identified, which supported 67% (18/27) of the original indicators about the data collection system in the preliminary framework and generated 9 new indicators. After comparison, 11 original indicators were further merged. A total of 16 indicators, including 10 facilitators and 6 barriers, were developed to measure the component data collection system (Multimedia Appendix 3).

#### Functions of the Data Collection System

A total of 17 interview codes were related to the subcomponent *functions of the data collection system.* They supported 8 original indicators of this subcomponent. Two new indicators emerged, and 5 original indicators were merged. A total of 5 indicators, including 3 facilitators and 1 barrier, were finalized to measure the subcomponent functions of the data collection system.

The practitioners agreed that the functions of the CRIMS should facilitate the visualization of routinely collected data. The CRIMS system should be humane, convenient, and error-free for data collection. For example, the system should remind data collectors wherever logic errors or incompleteness appear in data entry. In H304’s words, “Machine can do something for human beings.”

In recognition of the effect of *smart chart* and drop-down menus, some practitioners (C202, H302, and H305) suggested that the CRIMS should provide a user-friendly interface, allowing clinicians to add descriptive free text data; visualize data; and search by keywords, such as symptoms of a disease. The system should have convenient or automatic functions, such as iPhone’s one-click for all end users, and should eliminate tedious extra work. The hospital practitioners were not satisfied with the CRIMS menu allowing limited details. It was inconvenient and difficult for H303 to add additional text data:

*Some definitions are too narrow. For example, loss for follow-up could have a variety of reasons in reality, but we could not enter these data. Another example is the patient background. They may have lots of opportunistic infections without clinical symptoms; however,there are not enough options provided by the system to capture all situations.* [H303]

An information system without adequate functions may impair data quality. C301 spent nearly 15 minutes, one-third of her interview time, to elaborate on this topic according to her work experience. Ascertain definitions of data items in the system were not in accordance with those of the data collectors, which may lead to missing data or inaccurate data collection.

#### Integration of Different Information Systems

The interview transcripts supported 4 of the 7 original indicators that discussed the integration of different information systems. Four indicators, 2 facilitators and 2 barriers, were clarified for measuring this subcomponent.

Although the item “integration of different information systems” was not in the weighting table, the negative effects caused by the lack of integration of data across information systems were emphasized by practitioners from hospitals (H302, H303, H304, H305, and H201). Hospital information systems are internal systems that do not connect to external systems via the internet. Access to the CRIMS was only available on a few authorized computers in hospitals via internet connectivity. Clinicians could not read any information from the CRIMS beyond their hospital. Repetition in reporting often happened, causing *a wasted job* that could lead to clinicians’ reporting cards being “thrown into a rubbish bin” (H305). Therefore, it is a common sentiment that appropriate integration of the CRIMS with hospital information systems is needed.

In addition, 6 practitioners raised the importance of comprehensive data storage in the CRIMS information system (A101, A401, C106, H302, H303, H305). They suggested the system should include all work functions and topics, and cover all geographic regions from village, county, city to the province and national levels. From the national health administrator’s perspective (A401), the CRIMS should be such a system:

From the perspective of a specific case reporting system, I think, it is a very comprehensive system; maybe no other disease reporting system can be as comprehensive as it is. The AIDS (CRIMS) should be the most comprehensive one.

A401

#### Technical Support

A total of 12 codes identified from the transcripts discussed technical support. Two new indicators emerged and supported the original indicators in the preliminary framework. Three indicators, 2 facilitators and 1 barrier, were finalized for measuring this subcomponent.

Practitioners (C302, C202, C104, C105, and H101) stated that insufficient technical support could inhibit the quality of the CRIMS data collection process. They emphasized that technical support should also be available for data entry. Technical support differed from training. It should be available before and during data collection. Practitioners from the county CDCs (C104 and C105) recommended that technical support for data entry should include a multimedia-supported electronic network that stores frequently asked questions, allows end users to share experiences, and provides help to use the system. It should offer access to higher level support such as that from national institutions. Technical support must be comprehensive, problem-focused, and formal.

Technical support became exceptionally critical for a data collection task assigned by high-level authority without training. Given that data collection tasks were often directly deployed by the high-level authority through issuing an official notification (C104), A101 believed that a competent team leader could play a role in offering technical support.

#### Devices for Data Collection

Of the 5 original indicators about devices for data collection, 4 were supported and the other was merged. A new indicator emerged and was added. Four indicators, 2 facilitators and 2 barriers, were finalized to measure the subcomponent. The compatibility of the devices used for data collection with the CRIMS data collection system was a major concern.

The practitioners suggested that data collection devices should be of good quality (C106, C104, C102, and H101), reliable, fast, and fit for surfing the internet and should neither crash nor break down (C302 and C304). Prompts, such as “the system is under maintenance” (C102), were not welcomed by practitioners. They expected that the devices could help them perform their data work even at the peak time of data entry. It should be free from traffic jams (H306, H305, C203, and C102).

Regarding data backup and security, the CRIMS has specific policies requesting the duration of data storage and the frequency of data backup to mitigate the risk of data loss (H306).

## Discussion

### Principal Findings

This study used the expert elicitation research method to verify a preliminary 4D component framework for measuring the quality of the PHIS data collection process in the context of the Chinese HIV/AIDS information management system, the CRIMS. The 28 public health data management experts for the CRIMS, with varied work experience and roles in their organizations, provided insightful inputs to issues related to the quality of the data collection process. They agreed with the 4 main components derived from the literature [12]. They ranked and commented on the importance of the original subcomponents based on their perceptions of the CRIMS data collection process. The 302 codes identified from the interview transcripts supported 75.2% (112/149) of the original indicators. These results provided the basis for a validated 4D component framework that fits well with the preliminary framework.

The validated 4D component framework was an improvement on the preliminary version. New items were identified in the expert elicitation process and added to the subcomponents of the data collection environment. These were organizational policy, high-level management support, and collaboration with parallel organizations. A total of 46 new indicators were generated and integrated into the framework, showing a wide range of characteristics elicited from the specific research context.

The original indicator statements were further simplified, merged, or deleted based on the 3 data analysis scenarios. The number of indicators in the framework finally decreased from 149 to 116.

There were changes within the framework in the proportions of the indicators for the 4 main components. The proportion of the indicators of the data collection environment increased from 13.4% to 31.9%, that for data collection management decreased from 49.0% to 35.3%, and that for the data collection system decreased from 18.1% to 13.8%. There was no change in the proportion for data collection personnel (19%). The factors that affect the quality of the data collection process are multifaceted from the practitioners’ perspective.

### Lessons Learned

The detailed feedback from the participants provided deep insights into many issues related to the quality of the data collection process and matters that require ongoing negotiation and development to improve it.

Under data collection management, the methods and protocols with the third ranking among all subcomponents need to be well developed, uniform, and implemented by data collection personnel. Responses on quality assurance emphasized the importance and challenges of this area. In some cases, data collection protocols and quality assurance procedures were developed and issued by high-level management in public health, but frontline personnel were not involved. This might make the data collection protocol and methods not operable or unfeasible in the field. Strategies to improve data collection management should include the involvement of frontline public health data collectors, especially those in hospitals, in the design phase of data collection protocols and quality assurance procedures [29].

A friendly data collection environment is an indispensable component of a high-quality public health data collection process. Participants ranked leadership and training as the two most important items for this component. This is consistent with and corroborated by the International Standard Organization’s recommendation that the top management should “demonstrate leadership and commitment with respect to the quality management system” [30].

Various identified organizational issues complemented the subcomponent spectrum for the data collection environment. This included avoidance of data collection intruding unduly on health facilities’ operations, such as routine health services in hospitals. This also included the adequacy of communication between different organizations, such as health administration and hospitals, CDCs and hospitals, and between data collection staff and their superiors. Financial and logistical support for the data collection process appeared to be a major issue, as is the case for health care organizations in many countries [29,31-33]. If the level of support is inadequate or not suitably administered, data quality will deteriorate.

On the data collection personnel component, all practitioners agreed on the importance of work attitude, competence, and data audit skills. There was some variation in opinion regarding the difficulty of the data collection process. The priority placed by the management in a hospital that performs the data collection process can significantly affect performance [34]. The fabrication of data or negligence indicated a poor attitude, requiring action by managers and supervisors. *Burnout* exhibited by staff might appear after long-term work in data collection and would require remediation, especially in hospitals [34].

Work competence was considered as a *must-have* capability for frontline data collectors. In addition to data quality audit skills, there should be an understanding of the objective of data collection and the definition of data items. Increasing the number of competent staff would, in principle, help to improve the data collection quality, although a practical difficulty has been the high turnover of recruited contract staff following training [32].

The fourth component, the data collection system, is an area that is influenced by the continuing changes in the performance and availability of ICTs [35]. Functions in the system should facilitate the visualization of routinely collected data. The system should be humane for those who operate it and be convenient and error-free for data collection [35]. An inappropriate function in the system may impair data quality. For example, if the definition of data items in the system does not reflect the reality of the work undertaken, the results will be unconvincing.

As identified in the preliminary 4D component framework, insufficient technical support inhibits the quality of the PHIS data collection process [12]. Additional features suggested by participants included storage of frequently asked questions and shared experiences, help for staff using the system, and access to higher level support such as that from national institutions. They also saw a need for the integration of the data collection system with other information systems because disconnection may result in repetitive reporting and inappropriate use of resources [5,33].

A study contribution is that, for the first time, we confirmed that the 4D components provide a picture of the structure and operation of the HIV/AIDS data collection process in China. The findings suggested that the Chinese HIV/AIDS information management practice provided an effective validation case and enriched the field of the quality of the PHIS data collection process. Three new subcomponents—organizational policy, high-level management support, and collaboration among parallel organizations—were considered to influence the quality of China’s public health data collection process. This provided evidence to clarify the effect of the data collection environment on the quality of the CRIMS data collection process. The 4D framework also advocates the involvement of relevant stakeholders in data quality management. This provides an example to suggest the potential of using this framework for root cause analysis to investigate and identify the *real* factors behind poor data quality.

Although this study provides useful inputs to management decisions within the CRIMS and to negotiations with other parties on resources and requirements, it is reasonable to believe that the framework is also applicable to other settings, such as emerging infectious disease surveillance [36], general health care, education, and criminal justice.

### Comparison With Prior Work

The context of this investigation was the Chinese HIV/AIDS program. However, many of the issues identified in the 2 sources of validation, the CRIMS and China, are also echoed in other health care systems. Inadequate staff training for data collection and limited support were also reported in birth registration in the United States [37] and in antiretroviral treatment for HIV infection in South Africa [38]. Poor communication across the health care sector and between providers was found in Aboriginal cardiac rehabilitation in Australia [39]. A lack of data linkage and sharing in electronic immunization data collection systems was described in Canada [40]. Job fatigue was found in general practitioners in European countries [34]. Regarding the transferability or generalizability embedded in the findings, this validation study has achieved design validity via a well-organized and executed research process [26].

As there are few extant public health frameworks focusing on the quality of the data collection process, there is a genuine contribution that this research has made to fill a critical gap on this topic. The successful abstraction of the 4D framework components, subcomponents, and indicator statements demonstrates the need for qualitative research in a problem domain without known measurement methods. Therefore, this study has taken the right method and approach given the novelty of the research topic, despite its importance in ensuring public health data quality.

### Limitations

A potential limitation of this study is that a relatively small sample of experts participated in the interview, which may be small for statistical probabilistic generalizability. The control strategy was to use the theoretical sampling method, including all levels and types of participating organizations, personnel roles, and experts in the CRIMS system. This eventually brought data saturation for qualitative inquiry and provided comprehensive views of the HIV/AIDS data collection process in China. Given that the purpose of this study was to use a qualitative method to validate a preliminary conceptual framework, we have achieved our aim.

Although the number of indicators was reduced from 149 to 116, these indicators need further item reduction for ease of use in large-scale public health settings. This can be achieved by conducting quantitative questionnaire surveys with public health data management personnel at all levels. This will improve the validity of the 4D component framework and allow the reduction of measurement items to a manageable level.

### Conclusions

This qualitative study validated 4D components for the quality of the PHIS data collection process in the context of the Chinese HIV/AIDS information management system, the CRIMS. The findings demonstrate that data collection management, data collection environment, data collection personnel, and data collection system are key components that determine the quality of the Chinese HIV/AIDS data collection process. The 4D component framework was further modified into a new pool containing 16 subcomponents and 116 indicators. They can be further tested and judged by practitioners and researchers in future public health data quality assessment studies.

## Appendix

Multimedia Appendix 1 Original 4D components of the quality of the public health information system data collection process [<xref ref-type="bibr" rid="ref7">7</xref>, <xref ref-type="bibr" rid="ref12">12</xref>-<xref ref-type="bibr" rid="ref15">15</xref>].

Multimedia Appendix 2 Agreement with an item being a component or subcomponent and its importance rank for the quality of the Comprehensive Response Information Management System data collection process (N=28).

Multimedia Appendix 3 Indicators, including facilitators and barriers, in each subdimension of the 4 dimensions of the quality framework of the data collection process for public health information systems.

## Abbreviations

China CDC: Chinese Center for Disease Control and Prevention
CRIMS: Comprehensive Response Information Management System
ICT: information and communication technology
PHIS: public health information system
