# Supplementary material for: Validation of 4D Components for Measuring Quality of the Public Health Data Collection Process: Elicitation Study
Source: J Med Internet Res. 2021 May 10;23(5):e17240. doi: 10.2196/17240 (PMC8145089; doi:10.2196/17240)
Supplement: Multimedia Appendix 1 [file jmir_v23i5e17240_app1.docx]

Original 4D components of the quality of the public health information system data collection process [7, 12-15].

| Component | Description | Subcomponents | Application to this study |
| --- | --- | --- | --- |
| Data collection management | An administrative process by which data is acquired, validated, stored, protected, and processed [7, 13]. | Data collection system;  Quality assurance. | Concepts were adopted except that the subcomponent *data collection system* was renamed as *data collection protocol.* |
| Data collector | A data collector is a stakeholder who collects or supplies data for the PHIS, with whom the data user should build up and nurture a relationship [12]. | Staffing pattern; Skills and competence; Communication; Perception of data collection. | Concepts were adopted except that the component *data collector* was renamed as *data collection personnel*. |
| Information system | A combination of hardware, software, network infrastructure, and trained personnel [14]. | Functions and technical support; Integration of different systems; Devices. | Concepts were adopted except that the component *information system* was renamed as *data collection system* and the combined subcomponent *functions and technical support* was separated. |
| Data collection environment | The context for data collection. In a government context, a PHIS is directly responsible to legislative, regulatory, and policy directives [15]. | Training; Leadership; Funding. | Concepts were all adopted. |
