# Supplementary material for: Validation of 4D Components for Measuring Quality of the Public Health Data Collection Process: Elicitation Study
Source: J Med Internet Res. 2021 May 10;23(5):e17240. doi: 10.2196/17240 (PMC8145089; doi:10.2196/17240)
Supplement: Multimedia Appendix 2 [file jmir_v23i5e17240_app2.docx]

Agreement with an item being a component or subcomponent and its importance rank for the quality of the Comprehensive Response Information Management System data collection process (N=28).

| **Item** | **n** | **Rank (mean score)** |
| --- | --- | --- |
| **Data collection management** |  |  |
| Data collection forms | 27 | 3 (4.96) |
| Data collection management system | 22 | 9 (7.95) |
| Data assessment strategy | 27 | 10 (7.96) |
| Definition of client ^a^ | 1 | - |
| Pilot of data collection protocol ^a^ | 1 | - |
| **Data collection environment** |  |  |
| Leadership | 26 | 1 (3.92) |
| Training | 27 | 2 (4.33) |
| Organizational management policy | 23 | 8 (7.72) |
| High-level managerial support | 22 | 12 (8.50) |
| Collaboration among parallel organizations | 23 | 14 (9.65) |
| Social factors | 16 | 16 (12.19) |
| Funding ^a^ | 3 | - |
| Incentives for data collector and clients ^a^ | 1 | - |
| Client cooperation ^a^ | 2 |  |
| **Data collection personnel** |  |  |
| Work attitude | 28 | 4 (5.89) |
| Competence | 28 | 5 (6.14) |
| Data quality audit skills | 28 | 7 (7.71) |
| Demographics | 17 | 15 (11.94) |
| The number of professional staff ^a^ | 2 | - |
| **Data collection system** |  |  |
| Technical support | 25 | 6 (7.24) |
| Automatic functions | 20 | 11 (8.00) |
| Input devices | 19 | 13 (9.53) |
| Structure and operation of the system ^a^ | 2 | - |

^a^ : New items elicited from the elicitation session.
