# Supplementary material for: Validation of 4D Components for Measuring Quality of the Public Health Data Collection Process: Elicitation Study
Source: J Med Internet Res. 2021 May 10;23(5):e17240. doi: 10.2196/17240 (PMC8145089; doi:10.2196/17240)
Supplement: Multimedia Appendix 3 [file jmir_v23i5e17240_app3.docx]

Indicators, including facilitators and barriers, in each subdimension of the 4 dimensions of the quality framework of the data collection process for public health information systems.

| Component | Subcomponent | Facilitator (n = 82) | Barrier (n = 34) |
| --- | --- | --- | --- |
| Data Collection Management (28, 13) | Data collection protocol (16, 7) | 1. Data collection protocol is needed to guide data collection which is aim-focused, operable, and clearly understandable for frontline data collectors. ^a^ | 1. Different report format leads to duplication and unnecessary complexity. |
|  |  | 2. Data collection protocol can be an interpretive guidance or manual including data collection form, data definitions, guidelines on collating/aggregating data, data auditing procedures, as well as other steps of data collection, handling, analysis, and reporting. | 2. The requirements located at the back of a form are often ‘overlooked’ in form processing. |
|  |  | 3. A standardized and uniform data collection form should be used by all data collectors. | 3. The collected data lack adequate precision for meaningful interpretation. |
|  |  | 4. Data collection form is clear, readable, comprehensive, and unambiguous. ^a^ | 4. The data collection tools are frequently changed. |
|  |  | 5. The reporting form is based on the WHO guidelines, and is designed to fit in one page for ease of use. | 5. The numerator and denominator of an indicator is from different sources. |
|  |  | 6. Availability of definitions and requirements of data item at the back of) data collection forms for data collectors to verify. | 6. Notebooks are used instead of the standardized tools. |
|  |  | 7. Have a unique number for each form and register, along with an accurate document version number to eliminate confusion. | 7. Differences in purpose, resources, methods, and data assessment among programs. |
|  |  | 8. The wording of the questions including the options to the multiple-choice questions must be accurate, direct, understandable, and answerable. ^a^ |  |
|  |  | 9. The number of questions should be suitable and controlled within the allotted data collection time. ^a^ |  |
|  |  | 10. The questions for data collection are within ethical consideration. ^a^ |  |
|  |  | 11. Data collection methods are well developed, uniform, applicable and implementable. ^a^ |  |
|  |  | 12. The logbooks are kept at the health facilities for convenience of reference. |  |
|  |  | 13. Consult with the local users of the forms and tools to integrate their input in designing and revising data items and data collection methods. |  |
|  |  | 14. Perform data back-up regularly. |  |
|  |  | 15. Data collection is integrated into routine data flow. |  |
|  |  | 16. The observed differences between data collection methods are expected in certain circumstances. |  |
|  | Quality assurance (12, 6) | 1. Conduct a pilot to assess the need, instrument and procedure of data collection. | 1. No clearly identified and uniform mechanisms to address data quality challenges. |
|  |  | 2. Each data collection facility maintains an independent quality assurance program to ensure data accuracy. | 2. Data management responsibilities are not clearly assigned. |
|  |  | 3. Designated unit or full-time, experienced data clerks or registrars to audit data. | 3. A lack of ownership of tasks for data quality monitoring and evaluation, limited human resources for execution. |
|  |  | 4. Key monitoring, evaluation and data management responsibilities at the national level are defined. | 4. Lack standard way of tracking or reporting completeness regarding coverage of the data collection organizations. |
|  |  | 5. Have independent data auditor. | 5. No data cleaning. |
|  |  | 6. A single page data summary is configured as part of the PHIS application to prevent data elements from missing during data transmission. | 6. The data audit reports submitted to the national level do not contain information about the reporting unit. |
|  |  | 7. An automatic quality assurance/quality control system to identify duplications, discrepancies, outliers, and data entry errors. |  |
|  |  | 8. Site-specific data quality reports are automatically sent to the corresponding clinics for necessary verification. |  |
|  |  | 9. A minimum of 20% of the submitted records are randomly selected and all the data elements are verified by a staff member other than the initial data collector each month. |  |
|  |  | 10. The cycle from the initiation of data collection at the source data site to confirmation of receiving information from the relevant data storage site is completed consistent and timely in the maximum of three months duration. |  |
|  |  | 11. Availability of a diverse range of data quality assurance mechanisms including regular (such as quarterly) supervision, scorecard, data verification via phone call. |  |
|  |  | 12. Ensure logic, integrity, reliability, completeness, timeliness, accuracy, no under-report of data. a |  |
| Data Collection Environment (28, 10) | Leadership (7, 2) | 1. Management has a clear roadmap for the assignment and execution of the tasks before job starts. a | 1. Limited human and financial resources. |
|  |  | 2. During data collection, the management has strong capabilities to pushing the job forward and ensuring the data collection procedures to follow the required standard. a | 2. Lack of understanding about the importance of data collection and not attending supervision, auditing, training, meeting organized by the CDC. a |
|  |  | 3. The management has power to issue policies, clarify and assign duty and tasks, and provide financial and material support. a |  |
|  |  | 4. The managers are professionals with good understanding about the importance of the data collection tasks and recognizing the contribution of the involved staff. a |  |
|  |  | 5. Contribution of data collection personnel is recognized in terms of cost reimbursement or appraisal. |  |
|  |  | 6. Decentralizing leadership. |  |
|  |  | 7. Regular supportive supervision visits to districts and facilities. |  |
|  | Training (6, 1) | 1. Provide standardized, systematic, targeted, and mandatory training sessions. | 1. Inadequate training on information management. |
|  |  | 2. Training is focused on operational skills and knowledge for field data collection including the definition of data to be collected, data collection methods and procedures, and communication skill. a |  |
|  |  | 3. Use diverse training needs assessment strategies including in-class and field assessment mechanisms to identify health workers’ training needs and verify the effectiveness of the training on an annual basis. |  |
|  |  | 4.   An expertise task force is formed, and its recommendations are incorporated into the training program. |  |
|  |  | 5.   Provide effective, multi-mode training including interactive, problem-solving, and on-line sources. |  |
|  |  | 6.  Provide continuous, high-quality, on-job training and mentoring after the initial induction and competence assessment. |  |
|  | Funding (5, 1) | 1. Dedicated clerk to enter data for healthcare providers to reduce data management cost. | 1. Limited human resources and financial constrain may impede the implementation and maintenance of information technology infrastructure, such as server and network in a clinic. |
|  |  | 2. Investment in specific sentinel clinic sites can provide data assurance. |  |
|  |  | 3. Funding for devices and vehicles. ^a^ |  |
|  |  | 4. Funding to enable comprehensive data collection. ^a^ |  |
|  |  | 5. Compensation for participants. ^a^ |  |
|  | Organizational policy (3, 4) | 1. Availability of policies ensuring sufficient funding, human resource, and material support. a | 1. Data collection was set up as a part-time job. ^a^ |
|  |  | 2. Embody effective management and coordination. ^a^ | 2. Narrow workspace insufficient for data collection. ^a^ |
|  |  | 3. Built-in reward and bonus schemes to incentivize data collection activities. ^a^ | 3. Increased workloads did not have more funding. ^a^ |
|  |  |  | 4. The culture of ‘eating big-pot rice’. ^a^ |
|  | High-level management support (4, 1) | 1. Superior provides assurance including funding, policy, training, materials, reward and punishment schemes. ^a^ | 1. The more layers between superiors and frontline data collection facilities, the more difficult to execute the data collection tasks. ^a^ |
|  |  | 2. Certain level of autonomy placed on data collectors. ^a^ |  |
|  |  | 3. Importance attached to a data collection task grows with the increase of superior’s attention to the task. ^a^ |  |
|  |  | 4. Superiors clarify the workflow and responsibilities instead of only assigning tasks. ^a^ |  |
|  | Collaboration among parallel organizations (2, 1) | 1. Parallel organizations should coordinate, cooperate, and facilitate with data collection. ^a^ | 1. Quality of the data collected by parallel organizations, if without centralized coordination, can be poor. ^a^ |
|  |  | 2. A centralized organization to coordinate parallel organizations. ^a^ |  |
| Data Collection Personnel (17, 5) | Perception of data collection (3, 3) | 1. High acceptability of the data collection system and its data among data collection personnel. | 1. Data accuracy is just as important as data users treating patients. |
|  |  | 2. Responsibility/commitment and level of engagement of data collectors. ^a^ | 2. Data collectors do not have ownership of data collection tasks and do not treat the task as their job responsibility. |
|  |  | 3. Dedication to data integrity. ^a^ | 3. Data-related activities are often compromised due to high-time commitment and other competing priorities. |
|  | Skills and competence (5, 0) | 1. Have a competence-based framework listing the desired skill mix for data management. ^a^ |  |
|  |  | 2. Have received initial and ongoing training on basic knowledge of data collection and have contextual information. |  |
|  |  | 3. Have clear strategies to collect data including contacting the client, using the client-request alternative contact numbers, addressing cultural and language barriers. |  |
|  |  | 4. Have ability to check data accuracy. |  |
|  |  | 5. In addition to expertise, competence is multi-faceted including abilities of communication, organization, coordination, and writing. ^a^ |  |
|  | Communication (5, 0) | 1. Confidentiality is thoroughly explained to the case. |  |
|  |  | 2. Be empathetic, allow clients to tell their stories, and conduct interview in a conversational style. |  |
|  |  | 3. Sense and respect the language and cultural identity of the client. |  |
|  |  | 4. Establish rapport and ease client anxiety. |  |
|  |  | 5. Have strategies to address cultural and language barriers with the client. |  |
|  | Staffing pattern (4, 2) | 1. Address challenges related to lacking trained staff to carry out quality assurance responsibilities. | 1. High staff turnover causes missing data or inaccurate data. |
|  |  | 2. Have dedicated data entry clerks to collect data instead of clinical staff. | 2. Most positions for data collection are project-funded and fixed-term appointment, lacking dedicated human resources. |
|  |  | 3. Publish clearly defined schedule and tasks to ensure that all the tasks are carried out appropriately. |  |
|  |  | 4. Have adequate staff to cover all responsibilities, including monitoring and evaluation to improve data quality. |  |
| Data Collection System (10, 6) | Functions of the system (4, 1) | 1. The data collection system needs to be designed to be easy for use and without burden on health facilities. ^a^ | 1. Poor system flexibility prohibiting reporting of exceptional events; system irresponsive to changing needs of decision makers. ^a^ |
|  |  | 2. Allow entry of free text data considered useful and relevant by data collectors. ^a^ |  |
|  |  | 3. Automatic functions are available for data logic check, aggregation, extraction, and analysis. |  |
|  |  | 4. Use smart and advanced technology such as drop-down menus, cloud-based system, and computerized point-of-care health information systems. |  |
|  | Integration of different systems (2, 2) | 1.Compatible record linkage and integration are available between different data collection systems. ^a^ | 1. Data collection systems are incomplete, not integrated and unreliable. This creates the burden of a double data entry and reporting when data are captured in both paper-based and electronic systems. |
|  |  | 2. Data collection system is comprehensive in work functions and geographic distribution. ^a^ | 2. Extensive use of multiple vertical or parallel data reporting systems. |
|  | Technical support (2, 1) | 1. Mentors and supervisors assist data collection in addition to providing clinical support. ^a^ | 1. Poor and insufficient IT support, which is inadequate for maintaining and updating the data collection system. |
|  |  | 2. Dedicated person to provide technical support for data entry including data auditing, error report, and correction. ^a^ |  |
|  | Device for data collection (2, 2) | 1. Use computers to collect data. | 1. Lack standard practices for storage and maintenance of source documents or data in accordance with any confidentiality guidelines. |
|  |  | 2. Devices are compatible with the data collection system, enabling the system to be fully equipped, fast, stable, accessible, and usable. ^a^ | 2. Lack policy guidance on duration of data storage and the frequency of data back-up to protect against data loss. |

Note: ^a^ denoting a new indicator emerged from the expert elicitation results.
